# Assessment of anti-inflammatory tumor treatment efficacy by longitudinal monitoring employing sonographic micro morphology in a preclinical mouse model

**DOI:** 10.1186/1471-2342-11-15

**Published:** 2011-06-23

**Authors:** Sanjay Tiwari, Jan H Egberts, Olena Korniienko, Linda Köhler, Anna Trauzold, Claus C Glüer, Holger Kalthoff

**Affiliations:** 1 Division of Molecular Oncology, Institute for Experimental Cancer Research, Comprehensive Cancer Center North, University Hospital Schleswig-Holstein, Campus Kiel, Germany; 2 Medical Physics, Department of Diagnostic Radiology, University Hospital Schleswig-Holstein, Campus Kiel, Germany

## Abstract

**Background:**

With the development of increasingly sophisticated three-dimensional volumetric imaging methods, tumor volume can serve as a robust and reproducible measurement of drug efficacy. Since the use of molecularly targeted agents in the clinic will almost certainly involve combinations with other therapeutic modalities, the use of volumetric determination can help to identify a dosing schedule of sequential combinations of cytostatic drugs resulting in long term control of tumor growth with minimal toxicity. The aim of this study is to assess high resolution sonography imaging for the in vivo monitoring of efficacy of Infliximab in pancreatic tumor.

**Methods:**

In the first experiment, primary orthotopic pancreatic tumor growth was measured with Infliximab treatment. In the second experiment, orthotopic tumors were resected ten days after inoculation of tumor cells and tumor recurrence was measured following Infliximab treatment. Tumor progression was evaluated using 3D high resolution sonography.

**Results:**

Sonography measurement of tumor volume in vivo showed inhibitory effect of Infliximab on primary tumor growth in both non-resected and resected models. Measurement of the dynamics of tumor growth by sonography revealed that in the primary tumor Infliximab is effective against established tumors while in the resection model, Infliximab is more effective at an early stage following tumor resection. Infliximab treatment is also effective in inhibiting tumor growth growth as a result of tumor cell contamination of the surgical field.

**Conclusions:**

Clinical application of Infliximab is feasible in both the neoadjuvant and adjuvant setting. Infliximab is also effective in slowing the growth of tumor growth under the peritoneum and may have application in treating peritoneal carcinomatosis. Finally the study demonstrates that high resolution sonography is a sensitive imaging modality for the measurement of pancreatic tumor growth.

## Background

Pancreatic cancer is the fourth leading cause of cancer death. It is characterized by high metastasis, uncontrolled proliferation and resistance to almost all current therapies. Consequently the prognosis of this disease is poor. Surgery is the only curative treatment option and is often followed up with adjuvant systemic chemotherapy and/or radiation. However, only about 10% of patients can be surgically treated and for those patients where surgery cannot remove the entire tumor, chemotherapy with or without radiation therapy is the best option [1]. The median survival rate following curative resection is less than 21 months and for non-surgical intervention the five year survival rate is about 4% [2]. More effective drug therapies and the ability to assess their effects at the level of the tumor is crucial for improved patient survival rates.

Animal models play an important role in the development and evaluation of new cancer therapies. In particular orthotopic tumor models more closely resemble human tumors since the blood supply and adjacent tissue more closely mirror the tumor's microenvironment. We have previously described an orthotopic xenotransplant model in SCID mice for the adjuvant treatment of pancreatic carcinoma [3]. In this model a human pancreatic adenocarcinoma cell line is mixed with matrigel and injected orthotopically and the tumor is resected 10 days later. Administration of therapeutic agents can then be used to determine efficacy in preventing local tumor recurrence and metastases. Interestingly, very few metastatic lesions were identified when the tumor was non-resected, suggesting that inflammation associated with resection triggers the growth of metastatic cells [4]. This model has also been used to include neoadjuvant and extended neoadjuvant treatment settings prior to tumor resection [5].

For testing of efficacy of cytotoxic chemotherapeutic agents, endpoint determination of tumor weight of biopsy samples and histological analyses has been sufficient. This is because the primary mechanism of chemotherapeutic agents is to perturb the cell cycle during the cell division or mitotic phase resulting in induction of apoptosis or necrosis. However, newer therapies which target signal transduction pathways, such as anti-growth factor antibodies and small molecule tyrosine kinase inhibitors, may affect important pathways by delaying tumor progression. In the presence of continued treatment, different responses may be observed at different time points including zero growth, regression and/or resumption of growth. Therefore, the monitoring of therapeutic efficacy and assessing the potential utility of new agents is difficult with traditional endpoints measurements of tumor size.

Noninvasive imaging techniques are of considerable value in the study of drug efficacy since the temporal pattern of the complex dynamics of cancer growth can be monitored [6]. Ultrasound on the other hand is one of the major anatomical clinical modalities. Innovative high frequency sonography permits high resolution imaging of the micro anatomy and thus is suited to study micro morphological changes in small animals including tumor growth in mice. Because high-frequency sound waves require a medium for transmission, they travel far better through liquids or solids than through air; sonography, therefore, works well in the abdomen but not in the thorax. High resolution sonography therefore is a promising approach for the assessment of pancreatic tumor growth. Previously high frequency sonography has been used to detect orthotopic pancreatic tumor growth in a mouse model and tumor size correlated well with fluorescent optical imaging of tumor growth [7]. Detection of liver metastases also compared favorably with MRI, CT or optical bioluminescent methods [8-12]. Other mouse tumor models that have used high frequency ultrasound for monitoring tumor growth include ovarian, endometrial, prostate and bladder cancer [13-16].

We have previously shown that specific inhibition of TNF-alpha with Infliximab or Etanercept significantly reduces tumor recurrence, as determined by endpoint measurement of tumor weight and histological analyses [4]. The aim of this study is to assess high resolution sonography for the in vivo monitoring of drug efficacy in pancreatic tumor. We characterize tumor growth rate during primary growth and following tumor resection and demonstrate that sonographic determination of tumor volume is a sensitive tool for monitoring therapeutic efficacy. The study identifies differences in tumor growth rate in response to Infliximab and identifes potential applications of Infliximab in the clinical setting.

## Methods

### Orthotopic xenograft of human PDAC cells and tumor resection

Four-week-old female severe combined immunodeficient beige (SCID/bg) mice weighing 14 to 19 g were obtained from Harlan-Winkelmann. The mice were allowed to become acclimatized for 10 days and housed in a sterile environment, in which bedding, food, and water were autoclaved (Scantainer). Animal experiments and care were in accordance with the guidelines of institutional authorities and approved by the Ethics Committee for Animal Experiments at Christian-Albrechts-Universität-zu-Kiel [number, V 362-72241.121 (16-1/06) and V 312-72241.121-7 (6-1/07)]. Mice were anesthetized with i.p. injection of 80 mg/kg ketamine (Aveco Co., Inc.) and 10 mg/kg xylazine (Rugby Laboratories, Inc.). A small (f1 cm) lateral subcostal laparotomy was performed. One million PancTuII cells, suspended in 20 μL matrigel (BD Biosciences), were injected beneath the capsule of the pancreas and the abdominal wall and skin were closed.

In the first experiment tumor growth in two groups with (n = 11) and without Infliximab treatment (n = 9) was monitored and compared. Infliximab was administered i.p. (10 μg/g body weight) on days 3, 10, 17, after tumor cell inoculation.

In the second experiment, tumors were resected ten days after inoculation of tumor cells. Relaparotomy was performed and the tumor-bearing pancreas were carefully mobilized and resected by subtotal pancreatectomy as described by us in detail previously. All mice survived the resection procedure and were randomly assigned to two groups: Infliximab treatment animals (n = 11), with application of Infliximab on days 3, 6, 10, 13, 17, 20 after resection. Control mice (n = 11) were injected with an equivalent volume of 0.9% saline (200 μl) on the same days. All animals were sacrificed 23 days postoperatively, and organs and tumors were examined as described previously.

### High resolution sonography

During the imaging procedure, mice were anesthetized with 2% isoflurane vaporized in medical air. Animal temperature was monitored and maintained at 37 C with a heated imaging platform. Tumor volume assessments were made by high resolution sonography. Imaging was performed with a Vevo 770 small animal micro-imaging system (VisualSonics, Toronto, Ontario, Canada) using a 40 Mhz ultrasound probe with axial resolution of 40 μm and a 14.6 mm field of view. A transducer with central frequency at 25 MHz, providing axial resolution of 70 μm with a 20 mm field of view, was used for larger tumor imaging. The pancreatic tumor region of interest was selected by obtaining cine loops of ultrasound image and an image frame showing the tumor at it's largest transverse diameter was selected for analysis.

**Figure 1:**
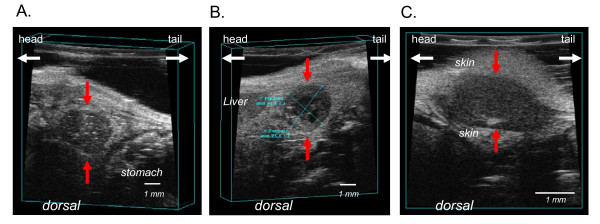
**Representative images of pancreatic tumor margins by ultrasound imaging**. Difference in echogenity define the tumor from the surrounding tissue. Tumors are hypoechogen (dark-grayish) compared to surrounding tissue. (A) primary orthotopic pancreatic tumor (red arrows). The light coloured specks (hyperechogenic) within the tumor may be microcalcifications or fatty deposits. (B) The cross-sectional area of the primary pancreatic tumor is depicted. The black areas (anechoic) within the tumor are fluid filled cysts correlating to necrotic areas. (C) Peritoneal tumor with surrounding skin.

Tumor volume was estimated by defining three-dimensional regions of interest using the VisualSonics image analysis software package. Two dimensional images were initially acquired at regular spatial intervals which were parallel and uniformely spaced at 100 μm. 3D image reconstruction was then performed automated by the Vevo 770 software (See http://www.visualsonics.com/vevo770 for additional information). Sonographic volume measurements were performed at days 7, 12, 17 and 21 in the first experiment and at days 10, 17 and 21 in the second experiment. Figure 1 shows representative ultrasound image of an orthotopic pancreatic tumor and peritoneal metastases.

### Physical measurements of volume and weight

At necropsy, visible tumors were dissected away from the normal pancreas and measured *ex vivo *by placing them on weighing scale.

### Statistical Analyses

All analyses were performed using the JMP software version 8.0 (SAS Institute, Cary, NC, USA). Multivariate Analysis of Variance (MANOVA) with repeated measures were used to identify interaction of group and temporal response and to study contrasts in temporal response during specific time periods. T-tests between groups were performed to compare the growth rates during various time intervals and also to test for differences at the final time point to compare the performance of the non-invasive imaging methods with the physical measurements of tumor weight and volume after sacrificing the animals. Average results per group are expressed as mean ± standard error of the mean unless noted otherwise. Figures showing statistical data were processed using Prism version 5 (GraphPad Software, La Jolla, CA, USA).

## Results

### Comparison of primary pancreatic tumor growth in response to Infliximab

**Figure 2:**
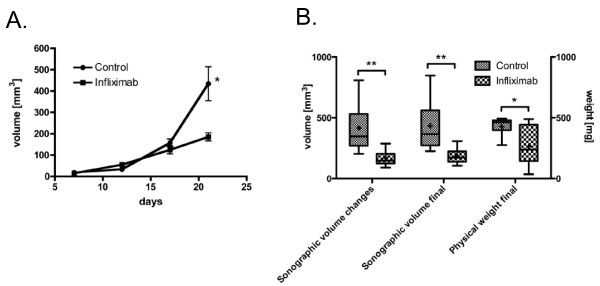
**Sonographic volume measurement and end-point tumor weight of the primary tumor in the non-resected model. **(A) Sonographic volume measurement and end-point tumor weight of the primary tumor in the non-resected model. Significance of group differences at each day was determined by 2-tailed student t-test (*: p < 0.002, n = 18). Measurements were performed on the respective day indicated following tumor cell inoculation. (B) Cross-sectional group differences for volume measurements by sonography (changes between day 7 and 21) or by physical determination of tumor weight (post mortem) n = 20. Box shows 25 - 75 percentile, whiskers 10 - 90 percentiles, + indicates mean and line the median. Significance was determined by 2-tailed Student t-test (*: p < 0.05, **: p < 0.005).

For the non-resected model, three dimensional sonographic volumetry was performed longitudinally to determine the effect of Infliximab on orthotopic pancreatic tumor growth. Analyses were carried out in those 7 control mice and 11 Infliximab mice (n = 18) that had no missing data for ultrasound measurements. Primary tumor volume was found to increase with Infliximab treatment but overall the increase was at a significantly slower rate than the untreated group (Figure 2A and MANOVA repeated measurements F-test p = 0.016). This is reflected by the overall increase in primary tumor volume of the Infliximab group from day 7 to day 21, which amounted to 41% of the increase of the control group (Figure 2B, 170 ± 19 mm^3 ^vs 416 ± 76 mm^3^, p = 0.015). In fact, up to day 17, the tumor volumes between the two groups were not significantly different but by day 21 a significant difference existed with Infliximab tumor volume at 43% of the volume in the control group (Figure 2A, 185.2 ± 19.0 mm^3 ^vs 434.6 ± 79.9 mm^3 ^p = 0.002).

To obtain insights into the kinetics of action of Infliximab, growth rate analyses were performed. An assessment of the growth rate revealed no significant difference from day 7 to day 12 but from day 12 to day 17 the increase in the rate of growth of Infliximab tumor was around three fifth of the untreated tumor (14.0 ± 3.2 mm^3^/day vs 24.7 ± 3.8 mm^3^/day, p < 0.05). From day 17 to day 21 tumor growth rate of the treated tumor was around one fifth (15.1 ± 4.3 mm^3^/day vs 69.4 ± 18.0 mm^3^/day, p = 0.003). Therefore Infliximab effect on growth rate is first observed 9 days after administration at a time when the tumor is established and palpable and it continues thereafter to be effective. Following sacrifice of the animals, a significant difference in the weight of the primary tumors was observed between the two groups (Figure 2B, 266 ± 48 mg for the Infliximab group versus 427 ± 29 mg for the control group, p = 0.03). The correlation of sonographically measured volume and physical measurement of weight was borderline significant (r2 = 0.2, p = 0.066) and was strongly influenced by one outlier (after exclusion of this outlier r2 = 0.31, p = 0.02). Immunohistochemical detection of pan-cytokeratin was performed on cryosections of excised pancreatic tumor tissue. Control tumors appeared to have a greater parenchymal tumor density than Infliximab treated tumor (See Additional file 1). The cellular composition of the stroma is currently under investigation.

### Comparison of tumor recurrence in response to Infliximab in the adjuvant treatment setting

**Figure 3:**
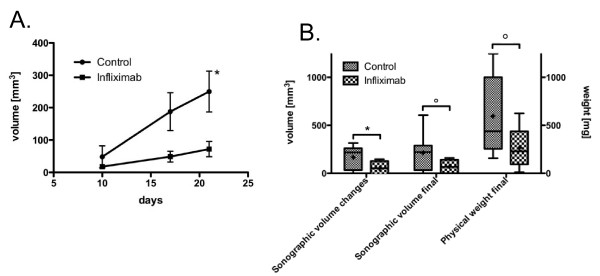
**Sonographic volume measurement and end-point tumor weight of the recurrent tumor in the resected model. **(A) Sonographic volume measurement and end-point tumor weight of the recurrent tumor in the resected model. Significance of group differences at each day was determined by 2-tailed student t-test (*: p < 0.05, n = 20). Measurements were performed on the respective day indicated following tumor cell inoculation. (B). Group differences for volume measurements by sonography, both longitudinally measured changes between day 7 and 21 and cross-sectional final volumes at day 21, compared to group differences by physical determination of tumor weight (post mortem), n = 16. Box shows 25 - 75 percentile, whiskers 10 - 90 percentiles, + indicates mean and line the median. Significance was determined by 2-tailed Student t-test (*: p < 0.05,: p < 0.1).

In experiment two, those 11 control mice and 9 Infliximab mice (n = 20) that had no missing data for ultrasound measurements were analysed to test whether the temporal development of the two groups differed. Indeed we observed a significant difference (Figure 3A and MANOVA, interaction of group and time p = 0.033). This is reflected by the overall increase in the Infliximab group from day 10 to day 21 which was 26% of the volume increase in the control group (54.2 mm^3 ^± 26.5 mm^3 ^versus 201.6 ± 48.2 mm^3^, p = 0.022). In contrast to primary tumor growth where differences in tumor volume were observed at the last timepoint, in the resected model the tumor volume of the treated group was 26% of the control group already at day 17 (Figure 3A, 48.8 ± 16.7 mm^3 ^vs 187.7 ± 58.5 mm^3^, p = 0.052). The gap widened somewhat more and at day 21 the difference was statistically significant (72.1 ± 23.4 mm^3 ^vs 249.5 ± 63.0 mm^3 ^p = 0.026).

To obtain insights into the kinetics of action of Infliximab, growth rate analyses was performed. Analyses revealed a significantly slower tumor growth rate for the Infliximab group between days 10 and 17 (4.4 ± 2.5 mm^3^/day vs 19.9 ± 5.1 mm^3^/day, p = 0.02). Subsequently although there was a difference in the mean growth rates for both groups between days 17 and 21 this was not statistically significant (5.83 ± 6.2 mm^3^/day vs 15.5 ± 11.3 mm^3^/day, p = 0.5).

For the comparison of the ability of weight measurements at the end of the study and noninvasive sonographical assessment of differences in volume at the end of the study to discriminate between the two treatment groups we could only analyse 16 animals: for 4 of the 20 animals the local recurrent tumor was not distinguishable from a secondary tumor growing from under the skin making weight measurements very inaccurate. This secondary tumor likely arose from tumor cell dissemination at the time of surgery. Tumor weight in the infliximab group was 44.5% of the weight in the control group (Figure 3B, 264.8 ± 88.3 mg vs 595.1 ± 124.9 mg, p = 0.09). In these 16 animals the volume difference measure by ultrasound showed a similar trend (Figure 3B, 71.0 ± 27.1 mm^3 ^vs 213.1 ± 56.6 mm^3^, p = 0.09) while the changes from day 10 to day 21 remained significant (Figure 3B, 49.7 mm^3 ^± 32.1 mm^3 ^versus 166.9 ± 37.1 mm^3^, p < 0.05). The correlation of sonographically measured volume and physical measurement of weight was highly significant (r2 = 0.4, p < 0.01).

### Comparison of the effect of Infliximab on postoperative surgical field

**Figure 4:**
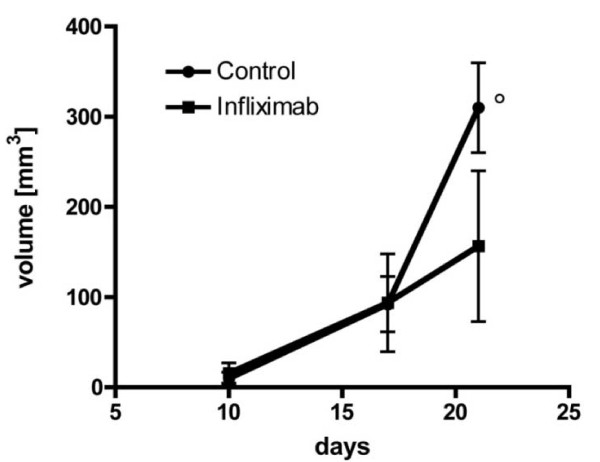
**Sonographic measurement of the volume of the peritoneal carcinomatosis in the resected model**. Significance of group differences at each day was determined by 2-tailed student t-test (: p < 0.12). Measurements were performed on the respective day indicated following tumor cell inoculation.

To determine if Infliximab is effective in inhibiting tumor growth as a result of tumor cell contamination at the site of surgical wound, the growth characteristics of peritoneal tumor was also studied. In the resected model peritoneal tumor was observed in 10 control mice and in 6 animals treated with Infliximab. Whereas at day 10 there was still some ambiguity about the presence of peritoneal tumors, at days 17 and 21 the spatial growth of this tumor in these mice were distinct from the primary recurrent tumor and allowed for accurate ultrasound quantification. As in the case with the resected tumor, high resolution sonography based quantification of peritoneal tumor volume revealed a different temporal growth pattern between controls and Infliximab treated groups between days 17 and 21 (Figure 4 and MANOVA repeated measurements F-test for interaction of time and group: days 10, 17, and 21: p = 0.06; days 17 and 21 p = 0.02). This was reflected by the difference in volume at day 21 between the two groups which showed a trend (156.6 ± 83.6 mm^3 ^for infliximab compared to 310.0 ± 49.8 mm^3 ^for the control group, p = 0.11).

A comparison of the temporal response patterns of the two groups revealed a significantly slower tumor growth rate for the Infliximab group from day 17 to day 21. The mean increase in peritoneal tumor volume was 15.7 ± 10.0 mm^3^/day in the group on Infliximab treatment compared to 54.4 ± 9.4 mm^3^/day for the controls (n = 16, p = 0.02). Therefore, Infliximab is effective in slowing the rate of peritoneal tumor growth at a time point when the tumor is established and larger.

## Discussion

If the survival rate for patients with pancreatic cancer is to improve, new agents which target specific cellular pathways and their effect in combination with other drugs needs to be explored. We have previously reported on the establishment of a resectable mouse model for pancreatic cancer for preclinical testing of adjuvant and neoadjuvant drug efficacy [3-5]. In this study we have utilized this mouse model to report on the non-invasive detection of pancreatic tumor growth in response to the TNF-alpha inhibitor Infliximab. Tumor progression was evaluated using a 3D high resolution sonography. We report here that longitudinal sonography measurement of tumor volume in vivo confirmed the inhibitory effect of Infliximab on primary tumor growth in both the resected and the non-resected models. End point differences in volume measured by sonography showed agreement with differences in tumor weight between the two groups measured post mortem. The findings indicate that for both primary and resected tumor models of pancreatic cancer, longitudinal ultrasound measurements can be used to reliably monitor therapeutic efficacy in vivo. Additionally, it permits to study the temporal development of tumor growth sequentially in a given animal providing more detailed insight into the dynamics of tumor growth and therapeutic effects.

Measurement of the dynamics of tumor growth revealed that in both models tumor growth continued to increase with Infliximab treatment but the rate of increase was less than that of the untreated tumor. For the primary tumor model reduction in growth rate was evident following 9 days of Infliximab administration. The reduction in growth rate was maintained throughout the duration of the experiment. A significant difference in tumor volume was first observed at the final time point on day 21. Therefore in the primary tumor model, Infliximab treatment was particularly effective in reducing tumor growth rate at the latter stages when the tumor is well established and palpable. In contrast, in the resection model, a significant reduction in tumor growth rate was observed following 7 days of Infliximab treatment (from day 10) which led to reduced tumor volume already at day 17 which was borderline significant. Between day 17 and 21, although the slower growth rate of the Infliximab group was not significant, tumor volume was still significantly less than the control at day 21. Longitudinal sonography thus allow to detect variable rates of tumor growth not accessible by post mortem measurements of tumor volume or weight. The findings indicate advantages for time dependent monitoring of tumor growth to study drug action and efficacy.

Local tumor cell dissemination associated with tumor resection and the inflammatory microenvironment following resection contributes to disseminated tumor cell proliferation and metastases. Sonography measurements in the resection model revealed a significant reduction in the growth rate of peritoneal tumors from day 17 with a trend for smaller tumor volume in the Infliximab treated group at day 21. The findings suggest that Infliximab would be effective as a postoperative treatment strategy for control of inflammatory reactions.

## Conclusion

In this study we have performed in vivo imaging to longitudinally monitor the growth rate of tumors in response to Infliximab which targets the TNF-alpha signalling pathway. In the setting of primary tumor growth and recurrent tumor growth we identify therapeutic efficacy of Infliximab to be associated with a 45 - 78% decrease in the growth rate of the tumor. We postulate that in such clinical settings, an agent will have therapeutic efficacy if it is able to stabilize the tumor growth rate. Although the failure to eradicate a tumor will not result in a cure, a cytoreduced tumor may nevertheless improve clinical outcome by not growing to a point where it becomes a deadly disease. In the resected model, Infliximab may be particularly effective at the early stages of tumor recurrence. We also show that Infliximab is also therapeutically efficacious against tumor growth in the postoperative surgical field. Finally we demonstrate that high resolution sonography is a sensitive imaging modality for the measurement of tumor growth.

The inhibition of signaling pathways as a therapeutic approach either by a single agent or in combination with chemotherapeutic agents can result in improved efficacy. In particular establishment of those time window during which different molecularly targeted agents are efficacious can lead to a novel paradigm of temporal targeting in cancer treatment. As more targeted therapies enter into clinical trials new models of proliferation and growth kinetics need to be established in order to obtain the true clinical potential of the drug. This can only be studied in vivo in longitudinal treatment studies employing molecular imaging or micro morphometric imaging approaches such as those investigated in this study. The establishment of reliable evaluation of anti-tumor efficacy in animal models is a prerequisite for improved success rate of emerging therapeutics in clinical trials.

## Competing interests

The authors declare that they have no competing interests.

## Authors' contributions

ST participated in data tabulation, analyses, interpretation of data and drafted the manuscript.

JHE planned the experiments, inoculated the mice with tumor cells, performed the surgical resection.

OK applied Infliximab to mice, performed ultrasound measurement and dissection of tumor. LK assisted in animal handling, dissection and ultrasound imaging. AT contributed to conception and design. CCG performed statistical analyses and helped to draft the manuscript. HK participated in conception, design and coordination of the project and helped draft the manuscript. All authors read and approved the final manuscript.

## Pre-publication history

The pre-publication history for this paper can be accessed here:

http://www.biomedcentral.com/1471-2342/11/15/prepub

## Supplementary Material

Additional file 1 **powerpoint, Immunohistochemical detection of cytokeratin using the pan-cytokeratin antibody KL-1 (AbCAM) on cryosections**. Figure containing immunohistochemical staining. Immunohistochemical detection of cytokeratin using the pan-cytokeratin antibody KL-1 (AbCAM) on cryosections. Immunohistochemical analysis was performed using the streptavidin-peroxidase technique and DAKO EnVision Systems (Dako Cytomation GmbH, Hamburg, Germany) according to the manufacturer's protocol. (Panel A) Four representative primary control tumors (untreated) depict a dense tumor parenchyma consisting of human tumor cells. (Panel B) Four representative Infliximab treated primary tumor showing less dense parenchyma but increased stromal density. Click here for file
