# Supplementary material for: Assessment of anti-inflammatory tumor treatment efficacy by longitudinal monitoring employing sonographic micro morphology in a preclinical mouse model
Source: BMC Med Imaging. 2011 Jun 23;11:15. doi: 10.1186/1471-2342-11-15 (PMC3143928; doi:10.1186/1471-2342-11-15)
Supplement: Additional file 1 — powerpoint, Immunohistochemical detection of cytokeratin using the pan-cytokeratin antibody KL-1 (AbCAM) on cryosections. Figure containing immunohistochemical staining. Immunohistochemical detection of cytokeratin using the pan-cytokeratin antibody KL-1 (AbCAM) on cryosections. Immunohistochemical analysis was performed using the streptavidin-peroxidase technique and DAKO EnVision Systems (Dako Cytomation GmbH, Hamburg, Germany) according to the manufacturer's protocol. (Panel A) Four representative primary control tumors (untreated) depict a dense tumor parenchyma consisting of human tumor cells. (Panel B) Four representative Infliximab treated primary tumor showing less dense parenchyma but increased stromal density. [file 1471-2342-11-15-S1.PPT]

## Slide 1
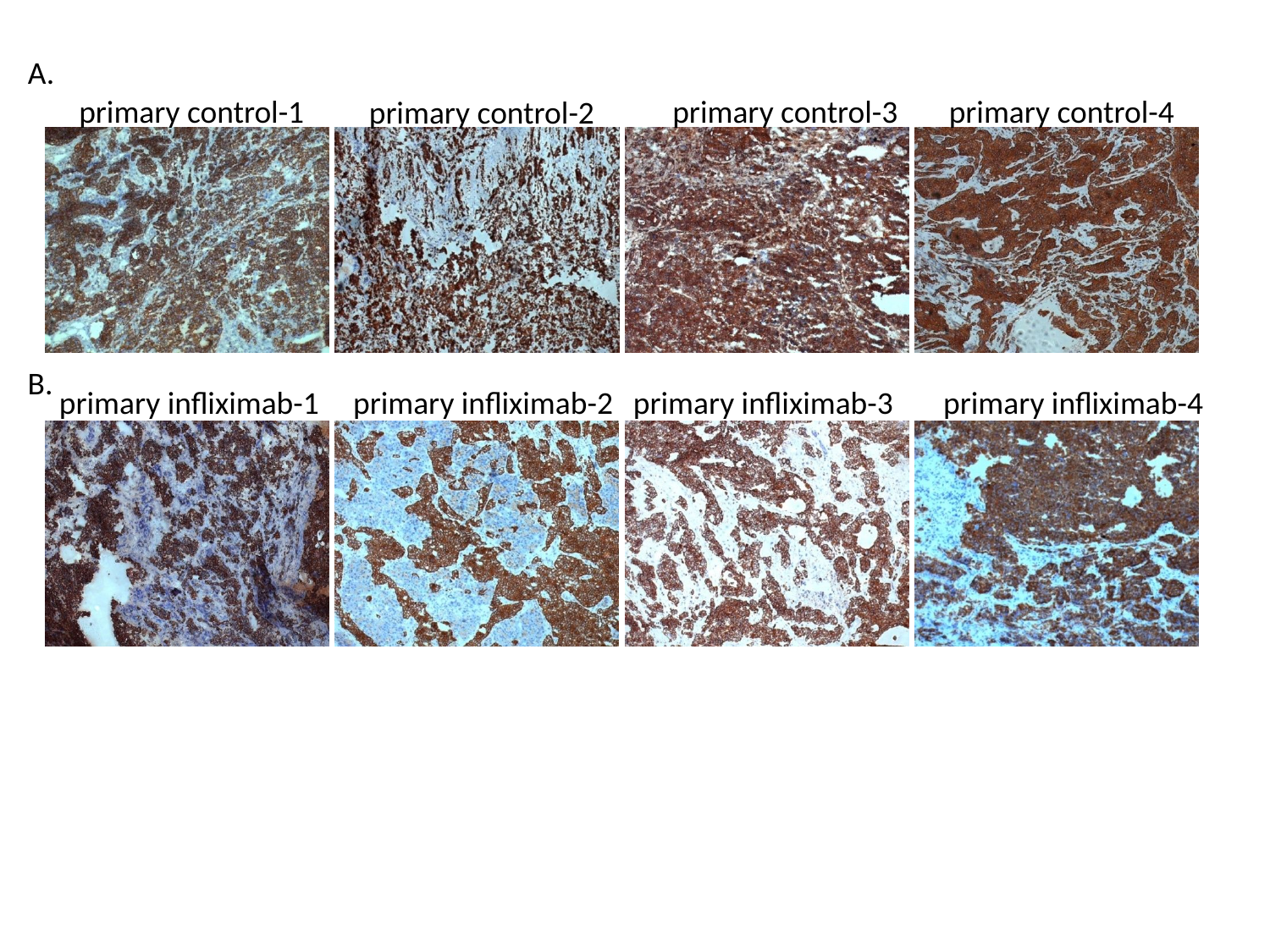

A.
primary control-1
primary control-3
primary control-4
primary control-2
B.
primary infliximab-1
primary infliximab-2
primary infliximab-3
primary infliximab-4
